# Dietary Patterns High in Red Meat, Potato, Gravy, and Butter Are Associated with Poor Cognitive Functioning but Not with Rate of Cognitive Decline in Very Old Adults^1^,^2^,^3^,^4^

**DOI:** 10.3945/jn.115.216952

**Published:** 2016-01-06

**Authors:** Antoneta Granic, Karen Davies, Ashley Adamson, Thomas Kirkwood, Tom R Hill, Mario Siervo, John C Mathers, Carol Jagger

**Affiliations:** 5 The Newcastle University Institute for Ageing,; 6 Institute of Health & Society,; 7 Human Nutrition Research Centre,; 8 Institute for Cell and Molecular Biosciences,; 9 School of Agriculture, Food and Rural Development, and; 10 Institute of Cellular Medicine, Newcastle University, Newcastle upon Tyne, United Kingdom

**Keywords:** dietary patterns, global cognition, attention, *apoE ε4*, very old adults, cohort study

## Abstract

**Background:** Healthy dietary patterns (DPs) have been linked to better cognition and reduced risk of dementia in older adults, but their role in cognitive functioning and decline in the very old (aged ≥85 y) is unknown.

**Objective:** We investigated the association between previously established DPs from the Newcastle 85+ Study and global and attention-specific cognition over 5 y.

**Methods:** We followed up with 302 men and 489 women (1921 birth cohort from Northeast United Kingdom) for change in global cognition [measured by the Standardized Mini-Mental State Examination (SMMSE)] over 5 y and attention (assessed by the cognitive drug research attention battery) over 3 y. We used 2-step clustering to derive DPs and mixed models to determine the relation between DPs and cognition in the presence of the dementia susceptibility gene.

**Results:** Previously, we characterized 3 DPs that differed in intake of red meat, potato, gravy, and butter and varied with key health measures. When compared with participants in DP1 (high red meat) and DP3 (high butter), participants in DP2 (low meat) had higher SMMSE scores at baseline (*P* < 0.001) and follow-ups, and better initial attention (*P* < 0.05). Membership in DP1 and DP3 was associated with overall worse SMMSE scores (β = 0.09, *P* = 0.01 and β = 0.08, *P* = 0.02, respectively) than membership in DP2 after adjustment for sociodemographic factors, lifestyle, multimorbidity, and body mass index (BMI). Additional adjustment for apolipoprotein (*apoE*) *ε4* genotype attenuated the association to nonsignificant in women but not in men in DP1 (β = 0.13, *P* = 0.02). Participants in DP1 and DP3 also had overall worse concentration (β = 0.04, *P* = 0.002 and β = 0.028, *P* = 0.03, respectively) and focused attention (β = 0.02, *P* = 0.01 and β = 0.02, *P* = 0.03, respectively), irrespective of *apoE ε4* genotype, but similar rate of decline in all cognitive measures over time.

**Conclusion:** DPs high in red meat, potato, gravy (DP1), or butter (DP3) were associated with poor cognition but not with the rate of cognitive decline in very old adults.

## Introduction

How to preserve cognitive function and reduce the prevalence and incidence of dementia in aging populations is of growing public health concern worldwide (1). Despite some evidence of declining age-specific rates of the most common subtypes of dementia, Alzheimer disease (AD)^11^ and vascular dementia (2), and intense investigation into the causes of age-related neurodegeneration, current pharmacological (e.g., immunotherapy) and nonpharmacological interventions (e.g., cognitive training) to slow or prevent cognitive impairment/dementia are limited (3, 4). Furthermore, risk factors for neurodegeneration in very old adults (aged ≥85 y)—who are at the highest risk of developing poor cognitive health (5)—are unknown (6). In the general population, advancing age, several genetic factors, notably *apoE ε4* genotype, as well as unhealthy lifestyles have been associated with an increased risk of dementia. For the latter, accumulated evidence points to the role of overall diet and of specific dietary components as modulators of cognitive aging and risk of neurodegenerative diseases (7–11). For example, inadequate consumption of healthy foods (e.g., vegetables and fish) (12, 13), excess intakes of specific nutrients (e.g., saturated and *trans *FAs) (14), and excess energy intake leading to obesity (15, 16) have been linked with more rapid cognitive decline and to neurodegenerative changes that lead to dementia in older adults (aged ≥65 y) (10, 11). Equally, diets rich in beneficial micro- (e.g., antioxidants) (17, 18) and macronutrients (e.g., ω-3 FAs) (13), food combinations (19), and healthy dietary patterns (DPs) derived from data at hand (20–23) or based on established dietary scores [e.g., Mediterranean-style diet (MeDi), Dietary Approaches to Stop Hypertension Trial (DASH), and combinations thereof] (24–27, 28–30, 31) may protect against progressive cognitive loss and dementia.

There is a growing interest in DP analysis in understanding the impact of nutrition on cognitive decline (32, 33) because DP is an approach that addresses the complexity of human diet and the likely synergistic effect of different foods and nutrients on individual health (34–36). Two main approaches to derive DPs have been used: a priori (or hypothesis driven) and a posteriori (exploratory or data driven) approaches (34–36). A priori developed DPs are based on predefined dietary scores for a specific DP (e.g., MeDi, DASH, or the Mediterranean-DASH Intervention for Neurodegenerative Delay diet) (24–31) or dietary guidelines (e.g., healthy eating index or healthy diet indicator), in which higher scores indicate healthier DPs, and are characterized by higher intakes of beneficial foods such as whole grains, fruits and vegetables, legumes, fish, and olive oil. The MeDi is recognized for its protective effect against cognitive decline and dementia (24–27) and other age-related diseases and conditions (37, 38). The a posteriori approach uses statistical methods such as factor or cluster analysis (39) to derive DPs and can take into account the total diet without the need for prior hypotheses about beneficial or detrimental effects of specific foods on health.

To date, evidence about the role of DPs in influencing age-related cognitive decline is inconclusive, leading to limited recommendations about how nutrition might be used to lower risk (7–11, 40, 41). Furthermore, few studies investigating the association between diet/DPs and cognitive decline or dementia have derived DPs empirically (33), or have considered the role of genetic risk factors in this association (19, 20, 24, 42), especially in very old adults (aged ≥85). To the best of our knowledge, the present study is the first prospective study to explore the association between DPs (derived a posteriori) and cognitive functioning and decline in very old men and women, and whether the relation may be changed by *apoE* status.

## Methods

### Participants

The Newcastle 85+ Study is a longitudinal study of over 1000 individuals born in 1921, who were recruited through general practices (GPs) in Newcastle and North Tyneside, United Kingdom as described elsewhere (43, 44). The study investigated a range of biopsychosocial factors that may affect physical and mental functioning of very old adults (aged ≥85) over a 5-y follow-up. At baseline (2006 and 2007), 851 participants consented to multidimensional health assessments (including diet and cognition) and GPs medical records review. Of those, 793 (93.1%) had a 24-h multiple pass dietary recall conducted on 2 nonconsecutive days by trained research nurses (45) at their usual place of residence (including care homes), and these dietary data were used to identify DPs as described previously (46). The analytic sample for the present study comprised 791 participants [302 (38.2%) men and 489 (61.8%) women] who had complete diet, health assessment, and GPs records data. Participants were followed up at 1.5 y (wave 2), 3 y (wave 3), and 5 y (wave 4) for health and cognitive outcomes.

### Ethics

The study was approved by the Newcastle & North Tyneside Local Research Ethics Committee 1. Written informed consent was obtained from participants or from a relative or caregiver if individuals lacked capacity to consent.

### Measurements

#### Cognitive assessments.

Global cognitive function was assessed using the Standardized Mini-Mental State Examination (SMMSE). The SMMSE is a brief dementia-screening instrument that provides a global score of cognitive function on a 0 to 30 points scale, with lower scores indicating worse performance. The assessment follows a standardized procedure for administration and scoring, and correlates well with the performance in activities of daily living (47, 48). A total of 788 (99.6%) out of 791 participants with complete health assessments (including diet) and GPs record review had baseline SMMSE scores. Global cognitive function was re-examined at 3-y (wave 3) and 5-y (wave 4) follow-up (range: 2.9–3.7 y and 4.4–5.6 y, respectively). SMMSE was not collected at 1.5-y follow-up (wave 2). A total of 463 (58.3%) participants completed the SMMSE at 3 y, and 328 (41.5%) at 5-y follow-up.

Attention was measured using the attention tasks within a reduced battery of the cognitive drug research computerized assessment system as described (49, 50). In brief, attention tasks comprised mean reaction times (speed scores) of correct responses (in milliseconds) for simple reaction time (SRT) measuring concentration and alertness, choice reaction time (CRT) examining similar tasks and accounting for additional information processing speed, and digit vigilance task (DVT) testing sustained attention while ignoring distractors. Three validated composite measures derived from these tasks were included: power of attention (PoA; sum of the 3 attention speed scores) measures focused attention; reaction time variability (RTV; sum of coefficients of variance of the 3 speed scores) examines attention fluctuation; and continuity of attention (CoA; combination of the accuracy scores from CRT and DVT) assesses sustained attention/attention accuracy over the testing period. For all attention measures except CoA, lower scores indicate better performance. A total of 746 (94.3%) participants with complete dietary data had at least 1 attention task score at baseline. Attention was re-examined at 1.5- and 3-y follow-up. Details about construction and validation of composite scores have been described previously (see supplemental material in 50).

The numbers of participants with complete diet and available cognitive test scores at each follow-up are summarized in **Supplemental Figure 1**.

#### Dietary assessment.

Dietary assessment and validation of 24-h multiple pass dietary recall in the Newcastle 85+ Study have been described elsewhere (45, 46). Briefly, trained research nurses evaluated habitual diet of study participants at baseline by taking a detailed record of foods eaten on the previous day on 2 different days of the week (except Fridays and Saturdays). About 5% of the study sample had their diets recalled by proxy. Over 2000 unique food codes were entered into a Microsoft Access–based dietary data system, and further grouped into 118 food groups based on McCance and Widdowson’s The Composition of Foods guidelines (46), and expressed as a 2-d mean value (in grams). These food groups were collapsed into 33 groups based on food and nutrient composition similarity and categorized as absent or present in each individual’s food intake (coded 0 and 1, respectively). A total of 30 of these food groups (i.e., excluding water, table sugar, and salad dressings, which were noninformative during clustering) were used in the cluster analysis as described (46) (**Supplemental Table 1**).

#### Covariates.

The following covariates from the literature were considered in multivariate analyses (1, 2, 20–24, 28, 29, 31): *1*) sociodemographic [sex; education (0–9, 10 to 11, ≥12 y); marital status (not married, married); social class (routine and manual, intermediate, higher managerial, and administrative professions)]; *2*) lifestyle [diet change in past year (yes, no); smoking (never, current smoker, former smoker); physical activity (low, moderate, high)]; *3*) health-related factors [number of chronic diseases (0–1, 2, ≥3); BMI (in kg/m^2^): underweight (<18.5), normal (>18.5–25), overweight (>25–30), obese (>30)]; and *4*) *apoE ε4* status (no *ε4* allele, 1+ *ε4* alleles) (46, 50). Chronic diseases (multimorbidity) from GP records included arthritis, hypertension, cardiac disease, respiratory disease, cerebrovascular disease, diabetes, and cancer (50). Diagnosis of dementia/AD was also determined from GP records.

### Statistical analysis

The SPSS 2-step clustering method used to derive DPs in this cohort and the associations between DPs and sociodemographic, health, and functional measures are described elsewhere (46). Briefly, the procedure creates small preclusters based on a log-likelihood distance criterion (for categorical variables) in step 1 and agglomerative hierarchical clustering to merge them into heterogeneous dietary groups in step 2. We used automatic selection and the Bayesian information criterion to determine the best DPs solution with 33 food groups, and by excluding food groups with consistently low importance factors. The smallest Bayesian information criterion (goodness of fit measure) was achieved with 30 food groups, which yielded a 3-DPs solution. The robustness and stability of the final DPs solution was re-evaluated by random ordering of cases and by comparing cluster solution characteristics (46).

#### Cognitive performance by DPs.

The differences in raw SMMSE and attention scores by DPs were compared using the Kruskal-Wallis test for ordered and nonnormally distributed continuous variables and the chi-square test for categorical variables (**Table 1**). All statistics were 2-sided at α = 0.05.

**TABLE 1:** Baseline characteristics and untransformed global cognitive and attention scores in very old adults by DPs over the study period^1^

Characteristics	All participants	DP1: high red meat	DP2: low meat	DP3: high butter	*P*^2^
Participants, *n*	791	276	260	255	
Sex, % (*n*)					
Women	61.8 (489)	57.6 (159)	64.6 (168)	63.5 (162)	
Education y, % (*n*)					<0.001
0–9	64.1 (501)	74.6 (203)	51.9 (134)	65.3 (164)	
10–11	23.4 (183)	19.1 (52)	25.2 (65)	26.3 (66)	
≥12	12.4 (97)	6.3 (17)	22.9 (59)	8.4 (21)	
Marital status, % (*n*)					0.02
Not married	69.4 (548)	66.9 (184)	76.0 (233)	65.5 (167)	
Married	30.5 (241)	33.1 (91)	23.9 (62)	34.5 (88)	
Social class, % (*n*)					<0.001
Routine/manual professions	51.1 (305)	58.1 (151)	39.4 (100)	56.1 (134)	
Intermediate professions	14.5 (109)	14.2 (37)	15.0 (38)	14.2 (34)	
Higher managerial/administrative	34.4 (259)	27.7 (72)	45.7 (116)	29.7 (71)	
Cognitive status by SMMSE					
Baseline, *n*	788	273	260	255	
Total SMMSE	26.1 ± 5.0	25.4 ± 5.7	27.3 ± 3.3	25.6 ± 5.4	<0.001
Impaired (≤25 SMMSE score), % (*n*)	27.2 (214)	34.4 (94)	18.1 (47)	28.6 (73)	
Normal (26–30), % (*n*)	72.8 (574)	65.6 (179)	81.9 (213)	71.4 (182)	<0.001
Follow-up at 3 y, *n*	463	144	166	151	
Total SMMSE	25.5 ± 5.4	25.1 ± 5.6	26.2 ± 4.8	25.1 ± 5.6	0.05
Impaired (≤25 SMMSE score), % (*n*)	33.3 (154)	38.9 (56)	25.9 (43)	35.9 (55)	0.04
Normal (26–30), % (*n*)	66.7 (309)	61.1 (88)	74.1 (123)	64.1 (98)	
Follow-up at 5 y, *n*	328	99	125	105	
Total SMMSE	24.9 ± 6.4	25.0 ± 6.0	26.2 ± 5.2	23.3 ± 7.6	0.01
Impaired (≤25 SMMSE score), % (*n*)	34.5 (113)	33.3 (33)	25.8 (32)	45.7 (48)	0.01
Normal (26–30), % (*n*)	65.5 (215)	66.7 (66)	74.2 (92)	54.3 (57)	
CDR attention battery					
Baseline, *n*	746	256	251	239	
SRT, ms	473 ± 492	510 ± 517	418 ± 149	493 ± 665	0.01
CRT, ms	648 ± 351	661 ± 305	606 ± 189	678 ± 496	0.02
DVT, ms	526 ± 70	534 ± 77	519 ± 66	523 ± 65	
PoA, ms	1613 ± 585	1683 ± 759	1541 ± 383	1614 ± 569	0.03
RTV	64 ± 20	66 ± 22	62 ± 17	65 ± 20	
CoA	52 ± 9	50 ± 10	53 ± 8	52 ± 10	0.01
Follow-up at 1.5 y, *n*	562	182	197	183	
SRT	488 ± 309	516 ± 378	431 ± 138	521 ± 356	0.02
CRT	663 ± 290	676 ± 332	611 ± 146	706 ± 348	
DVT	532 ± 73	537 ± 76	528 ± 65	532 ± 78	
PoA	1682 ± 594	1730 ± 682	1567 ± 286	1758 ± 717	0.04
RTV	64 ± 21	65 ± 23	61 ± 13	67 ± 25	
CoA	52 ± 8	53 ± 7	52 ± 9	52 ± 9	
Follow-up at 3 y, *n*	411	122	154	135	
SRT	476 ± 253	500 ± 312	458 ± 253	473 ± 184	
CRT	678 ± 371	678 ± 345	681 ± 461	675 ± 267	
DVT	534 ± 74	535 ± 78	536 ± 66	542 ± 78	
PoA	1688 ± 624	1713 ± 674	1667 ± 712	1690 ± 452	
RTV	63 ± 21	63 ± 19	63 ± 24	64 ± 19	
CoA	52 ± 9	52 ± 9	52 ± 9	52 ± 8	

1 All values are means ± SDs unless otherwise noted. SMMSE and CoA higher scores indicate better performance. SRT, CRT, DVT, PoA, and RTV lower scores indicate better performance. CDR, cognitive drug research; CoA, continuity of attention (expressed in CoA arbitrary units); CRT, choice reaction time; DP, dietary pattern; DVT, digit vigilance reaction time; PoA, power of attention; RTV, reaction time variability; SMMSE, Standardized Mini-Mental State Examination; SRT, simple reaction time.

2 Kruskal-Wallis test for ordered and nonnormally distributed continuous variables.

#### Global and attention-specific cognition by DPs.

SMMSE score was negatively skewed at each assessment (wave), and thus a transformed variable [NEWX = log_10_(30.5 − X)] was derived and used as a continuous variable in multilevel models. Lower transformed SMMSE scores reflect better cognitive performance (i.e., higher original scores). All attention reaction times were converted into seconds (s) and logarithmically (log_10_) transformed to correct a positive skew. PoA and RTV were also log_10_ corrected, whereas CoA was negatively skewed and was transformed as NEWX = 
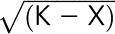
 (K = maximum score + 1). Lower transformed scores for reaction times (SRT, CRT, and DVT) and PoA, RTV, and CoA indicate better attention.

We used multilevel linear modeling (51) to *1*) examine the association between DPs and SMMSE scores at baseline, 3-y, and 5-y follow-up, and attention-specific tasks at baseline, 1.5-y, and 3-y follow-up [coded as 0 (baseline), 1 (1.5 y), 2 (3 y), and 3 (5 y)]; and *2*) identify baseline variables associated with the initial level and the rate of change in both global cognition and attention over the study period. This procedure examines simultaneously how each person changes over time and what predicts difference among people in their change. It accounts for variances in baseline cognitive level and in varying rate of change across waves. We fitted a series of linear growth curve models as follows: *1*) with time in study at within-person level (to examine the linear trend of time), and DPs at between-person levels [to test whether intercept (initial status) varied by DP], and with an interaction of DPs and time, to test for varying slopes (rate of change) by DP (Model 1); and *2*) with additional adjustment for baseline time-invariant confounders associated with cognition and diet [Model 2 (sociodemographic, lifestyle, and health-related variables) and Model 3 (*apoE ε4* genotype)] (**Tables 2** and **3**). All baseline time-invariant predictors (fixed effects terms) were categorical, and BMI (7.8% missing values) was imputed with sex-specific means. Random-effects terms included both intercepts and slopes of SMMSE and attention scores over time. We used SPSS mixed procedure (SPSS, 2002) with restricted maximum likelihood method and autoregressive error covariance matrix (AR1) to generate parameter estimates (β regression coefficients) with SEs for all outcomes. β-Coefficients for time represent the change in cognition/attention over time for the whole population (i.e., an average linear growth rate). β-Coefficients for DPs show the main cross-sectional effect of DPs on overall global cognition and attention, and β coefficient for time and DP interaction represents the change in cognition/attention over time attributable to DPs. The analyses for global cognitive decline by DPs were also stratified by sex.

**TABLE 2:** Multivariable adjusted β-coefficients of growth curve models for transformed SMMSE scores over 5 y by DPs in very old adults^1^

	Multivariable adjusted					
	Model 1^2^		Model 2^3^		Model 3^4^	
Outcome and effects/variable	β ± SE	*P*	β ± SE	*P*	β ± SE	*P*
SMMSE						
Entire cohort						
Time^5^	0.10 ± 0.03	<0.001	0.10 ± 0.03	<0.001	0.12 ± 0.03	<0.001
DPs^6^						
DP1 (high red meat)	0.16 ± 0.03	<0.001	0.09 ± 0.03	0.01	0.07 ± 0.04	0.08
DP2 (low meat) (ref)	0		0		0	
DP3 (high butter)	0.14 ± 0.03	<0.001	0.08 ± 0.03	0.02	0.03 ± 0.04	0.48
DP × time^7^						
DP1 × time	−0.01 ± 0.04	0.87	−0.003 ± 0.36	0.94	−0.02 ± 0.04	0.55
DP2 × time (ref)	0		0		0	
DP3 × time	1.37E-5 ± 0.04	1.0	0.01 ± 0.04	0.74	0.01 ± 0.04	0.90
Men						
Time	0.05 ± 0.04	0.28	0.05 ± 0.04	0.19	0.09 ± 0.04	0.04
DPs						
DP1	0.13 ± 0.05	0.01	0.10 ± 0.05	0.06	0.13 ± 0.06	0.02
DP2 (ref)	0		0		0	
DP3	0.07 ± 0.05	0.16	0.03 ± 0.05	0.61	0.01 ± 0.06	0.82
DP × time						
DP1 × time	0.04 ± 0.06	0.51	0.04 ± 0.06	0.48	−0.01 ± 0.06	0.89
DP2 × time (ref)	0		0		0	
DP3 × time	0.05 ± 0.06	0.48	0.07 ± 0.06	0.25	0.02 ± 0.07	0.75
Women						
Time	0.13 ± 0.04	<0.001	0.13 ± 0.03	<0.001	0.13 ± 0.04	<0.001
DPs						
DP1	0.17 ± 0.04	<0.001	0.06 ± 0.04	0.17	−0.01 ± 0.05	0.87
DP2 (ref)	0		0		0	
DP3	0.18 ± 0.04	<0.001	0.10 ± 0.04	0.02	0.02 ± 0.05	0.62
DP × time						
DP1 × time	−0.03 ± 0.05	0.56	−0.02 ± 0.05	0.69	−0.03 ± 0.05	0.53
DP2 × time (ref)	0		0		0	
DP3 × time	−0.02 ± 0.05	0.63	−0.02 ± 0.05	0.71	−0.01 ± 0.05	0.89

1 β-Coefficients ± SEs are estimates of fixed effects using transformed SMMSE longitudinal data [NEWX = log_10_(30.5 − X)]. Random-effects terms included both intercept and slopes of SMMSE scores over time. Time in the study was coded as baseline (0), 3-y follow-up (1), and 5-y follow-up (2). Increasing (positive) β indicates worse cognitive performance or decline. DP, dietary pattern; ref, reference; SMMSE, Standardized Mini-Mental State Examination.

2 Model 1 includes a linear trend of time, DPs, and their interaction term.

3 Model 2 is additionally adjusted for sociodemographic (sex, education, marital status, and social class), lifestyle (smoking, physical activity, and diet change in past year), and health-related variables (multimorbidity, BMI).

4 Model 3 is further adjusted for *apoE ε4* genotype.

5 The main effect of time across population over 5 y on SMMSE.

6 The main cross-sectional effect of DPs on overall (transformed) mean SMMSE score.

7 Change in SMMSE over time attributable to DPs.

**TABLE 3:** Multivariable adjusted β-coefficients of growth curve models for transformed attention tasks scores over 3 y by DPs in very old adults (entire cohort)^1^

	Multivariable adjusted					
	Model 1^2^		Model 2^3^		Model 3^4^	
Outcome and effects/variable	β ± SE	*P*	β ± SE	*P*	β ± SE	*P*
SRT						
Time^5^	0.02 ± 0.01	<0.001	0.02 ± 0.01	0.01	0.03 ± 0.01	0.002
DPs^6^						
DP1 (high red meat)	0.05 ± 0.01	<0.001	0.03 ± 0.01	0.003	0.04 ± 0.01	0.002
DP2 (low meat) (ref)	0		0		0	
DP3 (high butter)	0.03 ± 0.03	0.003	0.03 ± 0.01	0.02	0.03 ± 0.01	0.02
DP × time^7^						
DP1 × time	0.01 ± 0.01	0.55	0.01 ± 0.01	0.55	0.003 ± 0.01	0.73
DP2 × time (ref)	0		0		0	
DP3 × time	0.01 ± 0.01	0.49	0.01 ± 0.01	0.41	0.004 ± 0.01	0.52
CRT						
Time	0.02 ± 0.01	0.004	0.02 ± 0.01	0.01	0.02 ± 0.01	0.002
DPs						
DP1	0.03 ± 0.01	0.003	0.01 ± 0.01	0.21	0.01 ± 0.01	0.21
DP2 (ref)	0		0		0	
DP3	0.03 ± 0.01	0.003	0.01 ± 0.01	0.02	0.03 ± 0.01	0.01
DP × time						
DP1 × time	0.01 ± 0.01	0.62	0.01 ± 0.01	0.53	0.0002 ± 0.01	0.99
DP2 × time (ref)	0		0		0	
DP3 × time	0.01 ± 0.01	0.48	0.01 ± 0.01	0.47	0.01 ± 0.01	0.96
DVT						
Time	0.01 ± 0.003	0.03	0.01 ± 0.003	0.01	0.01 ± 0.003	0.03
DPs						
DP1	0.01 ± 0.004	0.01	0.01 ± 0.004	0.10	0.01 ± 0.01	0.15
DP2 (ref)	0		0		0	
DP3	0.002 ± 0.004	0.65	0.001 ± 0.004	0.84	−0.001 ± 0.01	0.76
DP × time						
DP1 × time	4.03E-5 ± 0.01	0.01	−0.0003 ± 0.004	0.95	−0.002 ± 0.01	0.61
DP2 × time (ref)	0		0		0	
DP3 × time	0.004 ± 0.01	0.36	0.003 ± 0.004	0.44	0.003 ± 0.01	0.48
PoA						
Time	0.02 ± 0.01	<0.001	0.02 ± 0.01	0.001	0.02 ± 0.01	<0.001
DPs						
DP1	0.03 ± 0.01	<0.001	0.02 ± 0.01	0.03	0.02 ± 0.01	0.01
DP2 (ref)	0		0		0	
DP3	0.02 ± 0.01	<0.001	0.02 ± 0.01	0.03	0.02 ± 0.01	0.03
DP × time						
DP1 × time	0.01 ± 0.01	0.23	0.01 ± 0.01	0.25	0.004 ± 0.01	0.59
DP2 × time (ref)	0		0		0	
DP3 × time	0.01 ± 0.01	0.24	0.01 ± 0.01	0.27	0.004 ± 0.01	0.60
RTV						
Time	0.01 ± 0.01	0.47	0.01 ± 0.01	0.29	0.01 ± 0.01	0.04
DPs						
DP1	0.03 ± 0.01	0.01	0.02 ± 0.01	0.09	0.02 ± 0.01	0.06
DP2 (ref)	0		0		0	
DP3	0.02 ± 0.01	0.03	0.02 ± 0.01	0.07	0.02 ± 0.01	0.15
DP × time						
DP1 × time	−0.002 ± 0.01	0.87	−0.004 ± 0.01	0.65	−0.01 ± 0.01	0.29
DP2 × time (ref)	0		0		0	
DP3 × time	0.001 ± 0.01	0.94	−0.001 ± 0.01	0.91	−0.01 ± 0.01	0.25
CoA						
Time	0.10 ± 0.07	0.17	0.12 ± 0.06	0.07	0.15 ± 0.07	0.04
DPs						
DP1	0.38 ± 0.09	0.05	0.20 ± 0.09	0.03	0.19 ± 0.10	0.07
DP2 (ref)	0		0		0	
DP3	0.16 ± 0.09	0.001	0.05 ± 0.09	0.60	0.0004 ± 0.10	0.1
DP × time						
DP1 × time	−0.03 ± 0.10	0.76	−0.03 ± 0.09	0.73	−0.06 ± 0.10	0.54
DP2 × time (ref)	0		0		0	
DP3 × time	0.03 ± 0.10	0.77	0.04 ± 0.09	0.64	−0.01 ± 0.10	0.89

1 β-Coefficients ± SEs are estimates of fixed effects using transformed longitudinal data for all attention tasks [all log_10_ transformed except CoA, which was transformed as NEWX = 
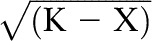
, K = maximum score +1]. Random-effects terms included both intercept and slopes of attention scores over time. Time in the study was coded as baseline (0), 1.5-y follow-up (1), and 3-y follow-up (2). Increasing (positive) β indicates worse attention or decline in attention. CoA, continuity of attention (expressed in CoA arbitrary units); CRT, choice reaction time; DP, dietary pattern; DVT, digit vigilance reaction time; PoA, power of attention; ref, reference; RTV, reaction time variability; SMMSE, Standardized Mini-Mental State Examination; SRT, simple reaction time.

2 Model 1 includes a linear trend of time, DPs, and their interaction term.

3 Model 2 is additionally adjusted for sociodemographic (sex, education, marital status, and social class), lifestyle (smoking, physical activity, and diet change in past year), and health-related variables (multimorbidity and BMI).

4 Model 3 is further adjusted for *apoE ε4* genotype.

5 The main effect of time across population over 3 y on attention.

6 The main cross-sectional effect of DPs on overall (transformed) mean attention scores.

7 Change in attention over time attributable to DPs.

All analyses were conducted using IBM SPSS (V.19 or 21; IBM Corporation, Armonk, New York).

### Sensitivity analysis

We compared participants lost to follow-up, through either withdrawal or death, with those still in the study 5 y after baseline by Mann-Whitney *U* test for ordered and nonnormally distributed continuous variables and by chi-square tests for categorical data (supplemental material).

Mixed models were further adjusted for sex-specific energy quartiles, supplement intake (yes, no), and number of medications (46, 50).

Cognitive status was also defined as normal (SMMSE raw scores ≥26) or impaired (SMMSE raw scores ≤25) (47, 48, 50) at baseline and 3- and 5-y follow-up. Incident cognitive impairment was defined as crossing the 25-point threshold of the SMMSE (47, 48, 50), and cognitive decline as a loss of ≥3 points from the baseline score (i.e., clinically meaningful or reliable change) (52) 3 and 5 y later. We used several logistic regression models to explore associations between DPs and prevalent or incident cognitive impairment or decline [OR (95% CI)] in participants with assigned DPs and baseline cognitive status (*n* = 788). Models were unadjusted (Model 1), adjusted for sociodemographic variables (Model 2), and adjusted for other lifestyle and health-related covariates in Models 3 and 4 (**Supplemental Tables 2** and **3**). Covariates with missing data were imputed to the reference values. Additional adjustments included sex-specific energy quartiles, supplement intake, and number of medications. The models were repeated in a sample without diagnosis of dementia/AD (from GP records) at baseline (i.e., 59 participants were excluded from the analysis) and in those living in the community (i.e., excluding 34 participants living in care homes).

Multicollinearity of confounders was assessed by examining the correlation matrix and multicollinearity diagnostics (i.e., tolerance, eigenvalues, and condition index).

## Results

We have previously reported 3 distinct DPs [high red meat (DP1), low meat (DP2), and high butter (DP3)] among participants in the Newcastle 85+ Study. These DPs differed in the participants’ consumption of 8 main food groups (i.e., butter, unsaturated fats spreads and oils, gravy, potato/potato dishes, red meat/meat dishes, legumes, coffee, and snacks), and the DPs were associated with differences in key sociodemographic, health, and functioning variables (46). Briefly, DP1 (high red meat) had the highest proportion of participants eating red meat/meat dishes, gravy, potato/potato dishes, legumes, and unsaturated fats spreads and the lowest proportion of participants consuming butter than participants in DP2 and DP3. In DP2 (low meat), participants did not consume a lot of meats (i.e., red and processed meats, bacon and ham, poultry), gravy, and potato and food moderate in butter and, when compared with others, ate the highest proportion of fruits, fish and seafood, nuts, whole grains and cereal products, low- and high-fat dairy, soups, and coffee. Therefore, the DP2 group was considered the healthiest and was used as a referent in present analyses. Unlike the participants in the other DP groups, participants in the DP3 (high butter) group ate moderate amounts of red meat, and food high in butter and low in other saturated and unsaturated fat spreads and oils (Supplemental Table 1) (46). A comparison of nutritional and blood biomarkers profile across DPs showed that DP1 had the highest percent energy (E%) from protein and starch, whereas DP3 had the highest food energy density, total fat, cholesterol, SFAs, MUFAs, and E% from fat. When compared with participants in DP1 and DP3, those in DP2 had favorable blood lipid profile (i.e., the lowest total cholesterol and the highest HDL cholesterol), and were more likely to be *apoE ε4* negative. They were also more advantaged in several socioeconomic indicators (i.e., higher education, social class, home ownership, and affluence of area of living), were healthier (i.e., the least likely to be disabled, obese, or depressed and to have dementia at baseline), and were more physically active (Supplemental Table 1) (46).

### Cognitive performance by DPs

In unadjusted models, participants in DP2 (low meat) had the highest SMMSE scores at baseline (*P* < 0.001) and 3-y (*P* = 0.05) and 5-y (*P* = 0.006) follow-ups and thus were the most likely to be classified as cognitively normal (26–30 SMMSE points) at all 3 assessments. They also had the best performance on STR (*P* = 0.01), CTR (*P* = 0.02), PoA (*P* = 0.03), and CoA (*P* = 0.01) at baseline, but not 3 y later compared with others (Table 1).

### Global cognition by DPs over 5 y

Using multilevel models with the entire cohort, we examined the association between DPs and SMMSE scores at baseline and at 3- and 5-y follow-ups. The β estimates of SMMSE showed a significant increase (i.e., poorer performance or cognitive decline) over 5 y (*P* < 0.001 in all models), especially in women (*P* < 0.001) (Table 2). Specifically, the linear growth rate for SMMSE increased by 0.12 log-transformed SMMSE points per unit of time in all participants and by 0.13 in women after adjustment for sociodemographic, lifestyle variables, number of chronic diseases, BMI, and *apoE ε4* genotype (Model 3 in Table 2), indicating cognitive decline with time.

Initial status (intercept) of SMMSE varied significantly by DPs in the entire cohort and separately in men and women (Model 1). In the model adjusted for sociodemographic, lifestyle, and health-related variables (Model 2), both DP1 (high red meat) and DP3 (high butter) were associated with overall worse SMMSE scores (β ± SE = 0.09 ± 0.03, *P* = 0.01 and 0.08 ± 0.03, *P* = 0.02, respectively) than DP2 (low meat). Among women, only DP3 was associated with worse overall global cognition (0.10 ± 0.04, *P* = 0.02). Adding *apoE ε4* genotype attenuated these associations to nonsignificant (Model 3), except for DP1 (high red meat) in men (0.13 ± 0.06, *P* = 0.02). However, in all analyses, the interaction term between DPs and time was nonsignificant, indicating that the slopes (rate of change) of log-transformed SMMSE means did not vary by DPs between individuals over 5 y.

In sensitivity analysis, the final model was additionally adjusted for sex-specific quartiles of energy, supplement intake (46), and number of medications, which did not change the results. The conclusions remained in analyses with a sample without dementia/AD diagnosis (at baseline) and in those living in the community (data not shown).

### Attention-specific tasks by DPs over 3 y

All attention reaction times showed a significant increase over time (i.e., slower or poorer performance) after adjustment for potential confounders (including *apoE ε4* genotype), indicating within-person decline over 3 y (Table 3). Specifically, mean SRT, CRT, DVT, PoA, RTV, and CoA increased (slowed) linearly by 0.03, 0.02, 0.01, 0.02, 0.01, and 0.15 log-transformed seconds per measurement occasion (~1.5 y), respectively (Model 3; all *P* ≤ 0.04).

In the fully adjusted model (Model 3 in Table 3), both DP1 (high red meat) and DP3 (high butter) were associated with overall slower reaction times in SRT (alertness/concentration) and PoA (focused attention/intensity of concentration). Specifically, the log-transformed means of PoA were slower by 0.02 for participants in DP1 (*P* = 0.01) and DP3 (*P* = 0.03) than for those in DP2. Worse performance in focused attention (PoA) among those in DP1 and DP3 was independent of participants’ poorer attention accuracy (CoA), indicating no intensity of concentration-accuracy trade-offs (Model 3) (50). Only DP3 was associated with worse overall scores in CRT (information processing speed), and no association was found for DVT (sustained attention) or RTV (attention fluctuation). For most attention tasks (except SRT and PoA), adding physical activity, BMI, and *apoE ε4* status to the initial model (Model 1) attenuated the association between DPs and attention outcome. For example, both DP1 and DP3 were associated with an increase in log-transformed RTV (β = 0.02, *P* = 0.01 for both), indicating greater fluctuation in attention compared with participants in DP2. However, the association was attenuated when adjusted for physical activity (data not shown). DP1 was associated with worse attention accuracy (CoA) in the model adjusted for lifestyle (Model 2), but was no longer significant after adjustment for *apoE ε4* status.

Additional adjustment for sex-specific quartiles of energy, supplement use, and number of medications or excluding participants living in care homes did not change the conclusions (data not shown).

Similar to the results for global cognition (SMMSE), the interaction term between DPs and time was nonsignificant, indicating that the slopes (rate of change) of attention scores did not vary by DP over 3 y. Convergence of the models could not be reached in sex-stratified analysis for most attention outcomes because of redundant covariance parameters.

### Results for sensitivity analysis

#### DPs and prevalent cognitive impairment (SMMSE).

We observed similar results as with multilevel models with transformed SMMSE (continuous) scores. Of 788 (99.6%) participants with baseline SMMSE scores and assigned DPs used in logistic regression models (Supplemental Table 2), 214 (27.2%) were classified as having impaired cognitive function (SMMSE ≤ 25). After adjustment for sociodemographic (sex, marital status, education, social class), lifestyle (smoking, dietary change in the past year), and health factors (BMI, multimorbidity) (Model 3), participants in both DP1 (high red meat) and DP3 (high butter) had increased odds of cognitive impairment than participants in DP2 (low meat) (OR: 2.30; 95% CI: 1.50, 3.54; *P* < 0.001 and 1.70; CI: 1.10, 2.64; *P* = 0.02, respectively). The association was no longer significant by additional adjustment for physical activity and depressive symptoms for those in DP3, but not for participants in DP1 (1.91; CI: 1.22, 3.01; *P* = 0.01), which remained significant after additional adjustment for sex-specific total energy, and *apoE ε4* genotype (1.81; CI: 1.08, 3.01; *P* = 0.02). Similar results were obtained when individuals with dementia/AD diagnosis (from GP records) were excluded from the models (*n* = 59) (Supplemental Table 3). DP1 was associated with a 74% increased risk of cognitive impairment (*P* = 0.03) compared with DP2 after adjustment for all covariates (Model 4), but it was attenuated by *apoE ε4* genotype (1.63; CI: 0.95, 2.79; *P* = 0.07) (data not shown). Adding supplement intake and number of medications to the models did not change the conclusions or when we excluded those residing in care homes from the models (*n* = 34) (data not shown).

#### DPs and incident cognitive impairment and decline over 3 and 5 y.

We used similar models as for the prevalent cognitive impairment to investigate the relation between DPs and incident cognitive impairment and decline. The incident impairment was defined as crossing a 25-point SMMSE cut-off, and decline as a loss of ≥3 SMMSE points (52) over 3- and 5-y follow-up. Significant associations between DPs and cognitive decline of ≥3 SMMSE were observed at 5-y follow-up (Supplemental Table 3) but not for incident cognitive impairment 3 and 5 y after baseline. DP3 (high butter) was associated with a 3.2-fold increased risk of cognitive decline (*P* = 0.001) in the fully adjusted model (Model 4), which was not changed by *apoE* status, sex-specific total energy, supplement intake, and number of medications in those free of dementia at baseline and residing in the community (data not shown).

## Discussion

Using multilevel models, we investigated the association between previously defined DPs (46) and global and attention-specific cognition in very old participants of the Newcastle 85+ Study. We found that DP1 (high red meat), a diet represented by a higher intake of red meat/meat dishes, gravy, and potato, or DP3 (high butter), a diet high in butter and low in unsaturated fat spreads/oils, was associated with worse overall attention in the very old, than DP2 (low meat), a diet low in red/processed meat, gravy, and potato and high in fish, fruits, nuts, dairy, and whole grain products. Specifically, participants in DP1 or DP3 had worse concentration (SRT), information processing speed (CRT), and focused attention (PoA) than participants in DP2, irrespective of *apoE ε4* status and other key covariates. On the other hand, the association between DP1 and DP3 and global cognition was attenuated to nonsignificant by *apoE ε4* genotype in the entire cohort and in women (DP3), but not in men (DP1). However, the magnitude of these associations was very small and may not convey clinical importance. In addition, the rate of cognitive change (both global and attention specific) was not affected, indicating that participants in all 3 DPs experienced similar rate of cognitive decline over 5 y, although those in DP2 had reached very old age in better cognitive form than others. When global cognitive decline was defined as a loss of ≥3 SMMSE points over 5 y (representing clinically meaningful change) (52), the risk was 3-fold higher in participants belonging to DP3 and was not reduced by *apoE ε4* status.

The extent to which DPs play a role in cognitive function and decline, and the risk of dementia, in the very old (aged ≥85) has not been investigated separately. Furthermore, the role of *apoE ε4* genotype in cognition-DP association is poorly understood. In addition, methodological differences in dietary assessments, DP derivation, cognitive tests, and definition of cognitive outcomes (e.g., impairment, decline, dementia/AD) across the studies preclude direct comparison of findings. However, certain similarities with our findings pertaining to more or less beneficial DPs (derived a posteriori) for cognitive health in late life may be highlighted.

For example, in the New York–based study of over 2000 older adults (mean age of 77.2 y) the middle and the highest tertiles of a DP that negatively correlated to red or organ meats and butter and positively correlated to fish, nuts, fruits, and cruciferous/green leafy vegetables were associated with 19% and 38% decreased risk of AD compared to the lowest tertile over 4 y and were not attenuated by *apoE ε4* status (19). This DP bears similarities with DP2, which had the highest percentage of participants consuming potentially more beneficial foods (e.g., fish, fruits, nuts, and whole grain and cereal products) and the lowest intake of red and processed meats (46). Participants in DP2 had the highest SMMSE scores at each assessment over 5 y and better initial attention. In the 3-City cohort study of over 8000 older French (aged ≥65), only *apoE ε4* noncarriers benefited from DPs characterized by daily intake of fruits and vegetables, and weekly consumption of fish, and had a 2-fold increased risk of AD if the consumption of n–6 PUFA rich-oils was not counteracted by n–3 PUFA food sources (20). In our study, men belonging to DP1 characterized by the highest intake of red meat/meat dishes, gravy (sources of SFAs and MUFAs), and unsaturated fats, spreads, and oils (sources of MUFAs and PUFAs) but low in fish/seafood (source of n–3 PUFAs) had overall worse global cognition irrespective of *apoE ε4* gene and other important covariates. This suggests that in this DP the negative effect of less healthy fats (e.g., SFAs) (14) in the presence of other beneficial nutrients (e.g., vitamin B-12) from red meat (53) and insufficient amounts of healthy FAs (e.g., PUFAs) (7–10, 13) from other food sources may predispose older adults to worse cognition regardless of genetic risk factors. Similarly, participants in DP3, which had the highest consumption of total fat, cholesterol, SFAs, and MUFAs (but not PUFAs) from mainly butter and red/processed meat and bacon/ham and not from unsaturated fats, spreads, oils, and fish (sources of PUFAs) were at a higher risk of cognitive decline (defined as a loss of ≥3 SMMSE points) over 5 y and had worse overall attention regardless of *apoE ε4*, total energy, number of medications, and dementia status at baseline compared with DP2 (sensitivity analysis). Therefore, when diet-cognitive health hypothesis is tested, a low or high consumption of a certain food or nutrient (a single food/nutrient approach) may reveal incomplete information without consideration of other foods/nutrients and their synergistic or antagonistic effect on the outcome (a whole diet/DP approach).

Several biological mechanisms may play a role in less favorable cognitive outcomes in older adults belonging to DP1 and DP3. For example, DPs high in foods rich in saturated fats may raise the risk of poor cognition by disrupting peripheral and brain lipid homeostasis, which in turn may affect neuronal membrane properties, synaptic plasticity, and signal transduction of neurons and increase the production of amyloid-beta (Aβ)—a hallmark of AD pathology (14, 54). Because the a posteriori approach accounts for a synergistic effect of other food groups and nutrients, this potential negative effect of saturated fats on cognition happens in the presence of more beneficial FAs (i.e., MUFAs in DP3) and in conjunction with other foods such as potato/potato dishes with high glycemic index (55) (i.e., DP1 had the highest E% from starch and carbohydrates) (46). This combination may in turn exacerbate fat-induced insulin resistance and decreased insulin sensitivity in the brain, and impaired clearance of Aβ peptide across the brain-blood barrier (56), to which the very old brain with excess vascular pathology may be particularly susceptible.

The results presented here should be interpreted with caution for several reasons. Although, to our knowledge, this is the first prospective evaluation of the DP-cognitive decline relation in the very old living independently and in care homes, diet was assessed only at baseline, which might not represent a stable diet over the study period. Seasonal variation of foods and exclusion of food recall on Fridays and Saturdays may have biased DP solution, and averaging 2-d recalls of food intake may have misrepresented habitual diet. Although we excluded those with dementia diagnosis (from GP records) in sensitivity analyses and confirmed the results, predementia states might have affected dietary choices before assessment of cognitive function. Prospective cognitive evaluation was limited to global cognition (i.e., SMMSE) and attention. Although we also considered a loss of ≥3 SMMSE points as a reliable, clinically relevant cognitive decline (52) in relation to DPs over 5 y, the log-transformed β estimates in mixed models were small and hard to interpret, and may not be clinically meaningful. Although the cognitive drug research attention battery has the ability to detect the change in attention with millisecond precision, the interpretability and the clinical relevance of detected change may be challenging. In a validation study (57), a decline of 59 ms in focused attention (PoA) over 6 mo in patients with a diagnosis of very mild AD (>26 Mini-Mental State Examination) and treated with cholinesterase inhibitors has been considered as clinically important. Participants in DP3 slowed by 144 ms (raw scores) in PoA over 18 mo (1.5 y) compared with 26 ms in those belonging to DP2, which may represent a clinically relevant decline. However, the rate of cognitive decline did not differ by DPs, possibly due to loss of power or selection bias. By 5-y follow-up we lost about half of our cohort (58), which might have introduced selection bias of very robust oldest-old survivals—affecting significant findings in cross-sectional analyses. The association between DP1 and DP3 and worse cognition may be contributed to uncontrolled confounding (e.g., oral health, food environment, and medication interaction). Finally, the results may have limited generalizability outside a white population of North European descent. The strengths of our study include its prospective design; representativeness of the United Kingdom population (including those in care homes); validated dietary assessment (45); robustness of clustering technique used to derive DPs, which considers whole diet (46); validated attention-specific cognitive measures (49, 50); and adjustment for several putative risks for cognitive decline/dementia including the *apoE ε4* status.

In conclusion, we have found the association between a posteriori–derived DPs (DP1, a high red meat, or DP3, high butter) and worse overall global cognition (in men) and attention after adjusting for several risk factors including *apoE ε4* genotype. However, the rate of cognitive decline was not affected by DPs. Diets high in red/processed meat, gravy, and potato/potato dishes or butter may predispose to cognitive impairment in very old adults. The finding needs to be further explored and validated in other prospective cohort studies to establish a causal link between a DPs dominated with foods rich in fats/cholesterol and cognitive function in very late life.

## Footnotes

11 Abbreviations used: AD, Alzheimer disease; CoA, continuity of attention; CRT, choice reaction time; DASH, Dietary Approaches to Stop Hypertension Trial; DP, dietary pattern; DVT, digit vigilance task; GP, general practice; MeDi, Mediterranean-style diet; PoA, power of attention; RTV, reaction time variability; SMMSE, Standardized Mini-Mental State Examination; SRT, simple reaction time.
